# Geographic and Racial Disparities in Access to Chimeric Antigen Receptor–T Cells and Bispecific Antibodies Trials for Multiple Myeloma

**DOI:** 10.1001/jamanetworkopen.2022.28877

**Published:** 2022-08-26

**Authors:** Raghad Alqazaqi, Carolina Schinke, Sharmilan Thanendrarajan, Maurizio Zangari, John Shaughnessy, Fenghuang Zhan, Guido Tricot, Frits van Rhee, Samer Al Hadidi

**Affiliations:** 1 School of Medicine, Mutah University, Karak, Jordan; 2 Myeloma Center, Winthrop P. Rockefeller Cancer Institute, University of Arkansas for Medical Sciences, Little Rock

## Abstract

**Importance:**

The use of chimeric antigen receptor–T cell (CAR-T) therapy and bispecific antibodies in multiple myeloma is expanding, with encouraging early results. It is unknown if the current geographic distribution of CAR-T therapy and bispecific antibodies in multiple myeloma allows access for patients in need, especially for Black populations, which have a higher incidence of multiple myeloma.

**Objective:**

To investigate if the current geographic distribution of CAR-T cell therapy and bispecific antibodies for multiple myeloma allows equitable access for Black patients with multiple myeloma.

**Design, Setting, and Participants:**

This cross-sectional study of data from CAR-T therapy and bispecific antibodies multiple myeloma clinical trials for all available studies listed in ClinicalTrials.gov until January 31, 2022. Only studies with 1 or more open sites in the US were analyzed. Data were analyzed February 2022.

**Results:**

A total of 162 clinical trials were found, and 69 analyzed—7896 participants were either enrolled or expected to enroll, with 4386 participants (55.5%) enrolled or to be enrolled in CAR-T therapies clinical trials. The vast majority of clinical trials (66 [96%]) were sponsored by industry, and there were 140 clinical trials sites. The mean number of sites per trial was 8.1 (7.8 for CAR-T trials [range, 1-30 trials] vs 8.7 for bispecific antibodies [range, 1-26 trials]). Only 35.9% of Black patients lived in a county with an open trial. For the 10 states with the highest proportion of Black residents (ranging from 18.6% to 41.4%), 6 of those states (60%) had no (3 states) or less than 3 clinical trial openings (3 states) for either a CAR-T or bispecific antibody study.

**Conclusions and Relevance:**

In this cross-sectional study, we found that the geographic distribution of clinical trials for CAR-T and bispecific antibodies may contribute to disparities in access to the most advanced clinical trials for new multiple myeloma therapies. Since most of the ongoing trials were sponsored by industry, regulating the distribution of clinical trial sites may reduce these inequities.

## Key Points

**Question:**

Is there equitable geographic access to chimeric antigen receptor–T cells (CAR-T) and bispecific antibodies trials for multiple myeloma in the US?

**Findings:**

In this cross-sectional study of data from 69 clinical trials in the US, 34% of the states analyzed had no CAR-T or bispecific clinical trial openings. There are limited open sites in states with the highest percentages of Black residents.

**Meaning:**

These results suggest that the current distribution of CAR-T and bispecific antibodies trials for multiple myeloma does not allow for equitable access for Black patients.

## Teaser

This cross-sectional study of US clinical trial data examines racial disparities in access to trials on chimeric antigen receptor–T cell and bispecific antibodies in treatment for multiple myeloma.

## Introduction

Outcomes for patients with newly diagnosed multiple myeloma have improved over the past decades.^1^ However, most patients will still relapse and require multiple lines of subsequent therapy. Patients with triple-class (proteasome inhibitor, immunomodulatory drug, and anti-CD38 monoclonal antibody) refractory disease have a particularly bleak outcome.^2^ The use of chimeric antigen receptor–T cell (CAR-T) therapy and bispecific antibodies in multiple myeloma is expanding with encouraging early results emerging for triple refractory patients from various clinical trials.^3,4,5^ Idecabtagene vicleucel is the first CAR-T product approved by the US Food and Drug Administration (FDA) for multiple myeloma patients who received at least 4 different prior lines of therapy.^6^ Ciltacabtagene autoleucel was shown to result in 97% overall response rate and 67% stringent complete response in patients with relapsed or refractory multiple myeloma in clinical trials and is currently approved by the FDA.^4^ Similarly, early exciting results indicate the teclistamab, a bispecific antibody, is a promising treatment option with 58% of relapsed or refractory multiple myeloma patients achieving a very good partial response or better.^5^

Disparities affecting Black patients with multiple myeloma include delayed diagnosis, lower use of novel agents including proteasome inhibitors, and lower utilization of palliative care.^7,8^ Black patients do not enroll in clinical trials at the same rate as non-Hispanic White patients and have low rates of use of novel therapies and autologous stem cell transplantation (ASCT).^7,9^ We previously reported^10^ that a disproportionally low number of Black individuals with hematological malignant neoplasms have been treated with approved CAR-T products. We hypothesized that one reason for this disparity is that Black persons do not live in states where these trials are being launched and herein conducted a cross-sectional analysis of the geographic distribution of CAR-T and bispecific antibody trials for multiple myeloma.

## Methods

Data on clinical trials were obtained from ClinicalTrials.gov, the largest clinical trials registry database that provides data on clinical trials that are completed or in process. We searched ClinicalTrials.gov in January 2022 using the terms *multiple myeloma*, *plasmacytoma*, *plasma cell dyscrasia*, *CAR-T*, *chimeric antigen receptor T cells*, *chimeric*, *bispecific antibodies*, *bispecific*, *BCMA*, and *T-cell engager*. We included all available trials with a listed status of completed, recruiting, active-nonrecruiting, terminated, or suspended. The collected data abstracted from ClinicalTrials.gov included study titles, National Clinical Trial identification numbers, trial phase, and intervention, actual or expected number of participants (in studies that did not complete enrollment), primary outcomes, recruiting sites, funders, and specific inclusion and exclusion criteria. ClinicalTrials.gov identifies 4 types of funders: US National Institutes of Health, other US federal agencies, industry, and all others (including individuals, universities, and community-based organizations). Funding support may include activities related to design, implementation, data analysis, or reporting. In our analysis, funding was identified as either industry or nonindustry. 2020 US Census Bureau data was used to obtain data on race and ethnicity.

The study was deemed exempt from institutional review board review by the University of Arkansas for Medical Sciences since data were publicly available. The study followed the Strengthening the Reporting of Observational Studies in Epidemiology (STROBE) reporting guideline for cohort studies. Analysis for this study was performed using R version version 4.1.2 and R Studio version 1.1.423 (R Project for Statistical Computing).

## Results

A total of 162 clinical trials were found in our review (Table 1). Only studies with 1 or more open sites in the US (69 studies) were analyzed. The majority consisted of phase I (41 studies [59%]) or phase I/II (14 [20%]) studies. There were 44 (64%) CAR-T therapy trials. A total of 7896 participants were either enrolled or expected to enroll in those clinical trials, with 4386 participants (55.5%) enrolled or to be enrolled in CAR-T therapies clinical trials. Every clinical trial had 1 or more recruitment site. Forty-five clinical trials (65%) were only open in the US while 24 clinical trials (35%) were open in the US and other countries. A total of 25 studies (36%) involved bispecific antibodies (Table 2). Studies were reported as investigator initiated in 47 clinical trials (68%), industry in 19 trials (28%), and governmental in 3 trials (4%) (Table 1). The vast majority of clinical trials (66 [96%]) were sponsored by industry. The primary outcomes of the analyzed studies were safety related, efficacy related, and both safety and efficacy related in 41 (59%), 11 (16%), and 16 trials (23%), respectively.

**Table 1.** Characteristics of CAR-T and Bispecific Antibodies Clinical Trials

Characteristics	Trials, No. (%) (N = 69)
Study phase	
I	41 (59)
I/II	14 (20)
II	10 (14)
III	3 (4)
Funding	
Industry	66 (96)
Nonindustry	3 (4)
Geographical distribution	
US alone	45 (65)
US and a global site^a^	24 (35)
Primary outcome reported	
Safety-related outcome	41 (59)
Efficacy-related outcome	11 (16)
Safety and efficacy–related outcome	16 (23)
Missing	1 (1)
States	
With open trial	34 (67)
With no trial opening	17 (33)

Abbreviation: CAR-T, chimeric antigen receptor–T cell.

^a^Global sites located in Europe, Canada, and China.

**Table 2.** Status of Included CAR-T and Bispecific Antibodies Clinical Trials

Characteristics	Studies, No. (%)	
	CAR-T	Bispecific antibody
US trials	44 (64)	25 (36)
Trial status		
Recruiting	25 (57)	15 (60)
Active, nonrecruiting	14 (32)	5 (20)
Terminated	0	3 (12)
Completed	4 (9)	1 (4)
Other/withdrawn	1 (2)	1 (4)

Abbreviation: CAR-T, chimeric antigen receptor–T cell.

One hundred forty unique study sites for the 69 analyzed clinical trials were identified. The mean number of sites per trial was 8.1 (7.8 for CAR-T trials [range, 1-30 trials] vs 8.7 for bispecific antibodies [range, 1-26 trials]). Multiple study sites had multiple different open trials (range, 1-19 open trials). Study sites were distributed in 34 different states. Most were Southern states (56 sites [40.0%]), followed by Northeastern (30 [21.4%]), Midwestern (29 [20.7%]), and Western states (25 [17.8%]). The mean number of trials per state was 8.68 (range, 0-37 trials). Seventeen states had no open CAR-T or bispecific clinical trials. Those include 4 in the Northeast (Maine, New Hampshire, Rhode Island, and Vermont), 4 in the South (Delaware, District of Columbia, West Virginia, and Mississippi), 2 in the Midwest (North and South Dakota), and 7 in the West (Idaho, New Mexico, Montana, Nevada, Wyoming, Alaska, and Hawaii) (Figure; eTable in the Supplement). The highest numbers of study locations were in New York (12 locations), Florida (11), and Texas (10) while the highest numbers of open studies were in New York (37 locations), California (34), and Texas (30).

**Figure.**
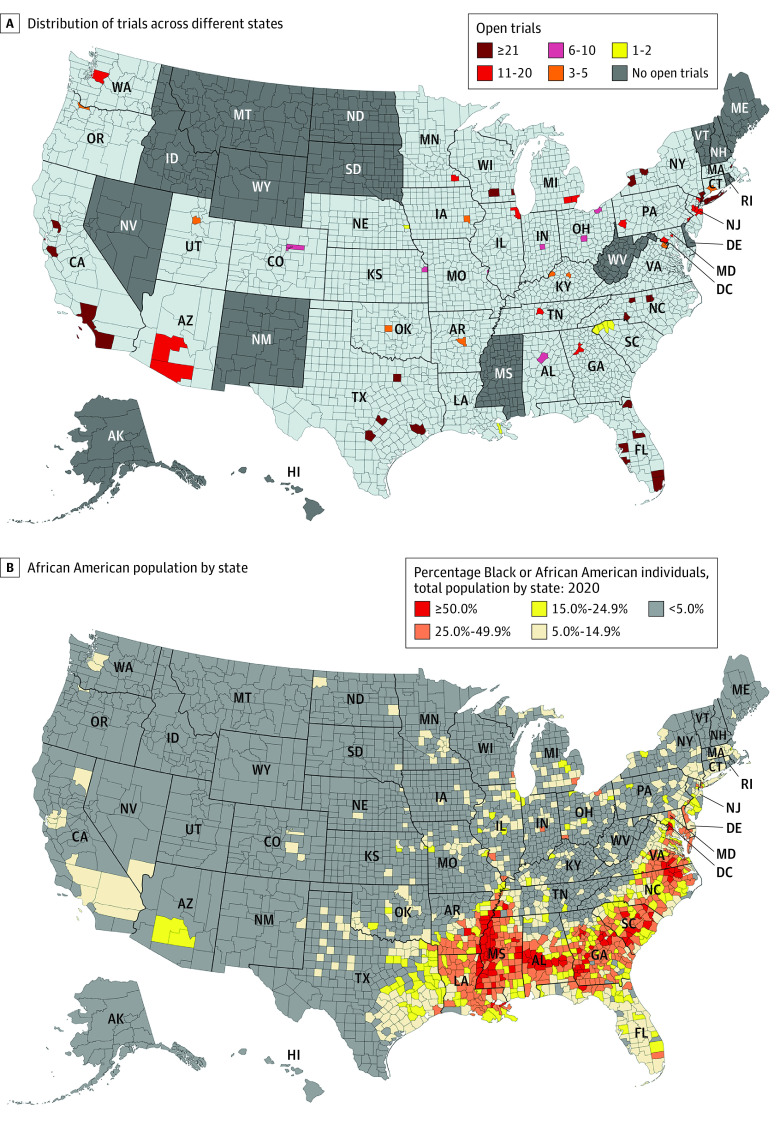
Distribution of Chimeric Antigen Receptor T Cells and Bispecific Antibodies Clinical Trials in the US

Using Census Bureau data, we found that only 35.9% of Black individuals (14 791 209 of 41 104 200) lived in a county with a CAR-T open trial. For the 10 states with the highest proportion of Black residents (range, 18.6%-41.4%), 6 of those states (60%) have no (3 states) or less than 3 clinical trial openings (3 states) for either CAR-T or bispecific antibody studies (Figure). Only 10 states (20%) had 50% or more of the Black population living in a county with an open CAR-T or bispecific antibody trial; only 21 states (41%) had 30% of their Black residents living in a county with an open CAR-T or bispecific antibody trial.

Because the administration of CAR-T and bispecific antibodies is usually done at a transplantation center, we investigated if trial distribution was associated with the number of patients in each state who had a ASCT done for multiple myeloma using Center for International Blood and Marrow Transplant Research data. The highest numbers of ASCTs for multiple myeloma were performed at the University of Arkansas for Medical Sciences (1283 transplantations), MD Anderson Cancer Center in Texas (1182 transplantations), City of Hope in California (1158 transplantations), Emory University in Georgia (1146 transplantations), and Mayo Clinic Rochester in Minnesota (1145 transplantations) (eTable in the Supplement).

## Discussion

We found that there were 17 states (34%) with no CAR-T or bispecific clinical trials open and that there were limited open sites in the states with the highest percentage of Black residents. For states with relatively higher Black populations, open trials tended to be available in counties with lower proportions of Black residents. These disparities in access to clinical trials for new multiple myeloma therapies was not associated with trial feasibility or availability of infrastructure because most of the states have at least 1 transplantation center (Table).

Most CAR-T and bispecific antibodies clinical trials were early phase clinical trials. Nevertheless, initial reports of their activity are promising. Currently they are used or studied in patients with multiple myeloma who have received multiple other types of therapy, although multiple clinical trials are currently under way using them as early treatments for multiple myeloma.

Currently, there is a high demand on CAR-T cell therapy with limited availability. Potential solutions can help in improving access to CAR-T cell therapy.^11^ This includes the development of new CAR-T products, efforts to increase existing production capabilities, development of off-the-shelf allogeneic CAR-T products, and future outpatient CAR-T product administration. The immediate availability of bispecific antibodies may help alleviate the constraints on CAR-T cell therapy manufacturing, and this can potentially help in opening more clinical trial sites to allow for better access to Black patients with multiple myeloma.

Enrollment of Black patients in clinical trials that resulted in CAR-T product approvals in the US for all hematological malignant neoplasms including multiple myeloma is suboptimal.^10^ Future efforts should aim to enroll proportions of Black patients that are similar to the demographics of incident cases of the studied cancer. Implementing the least acceptable race-specific percentage for enrollment of Black patients in clinical trials can be entertained to help in opening clinical trials in counties or states with no or limited access to clinical trials in an effort of increasing the enrolled Black patients in cancer clinical trials.^12^ The ideal number of Black patients to be enrolled in any clinical trial should aim for a proportion of Black participation that is similar to their proportion in incident multiple myeloma cases. We believe that the lowest acceptable race-specific percentage should be a new bar that clinical trials need to overcome before drug approval. Our analysis may help provide investigators and industry stakeholders interested in improving access to clinical trials for Black patients with multiple myeloma to focus their efforts on sites with higher percentages of Black residents with no access to clinical trials.

In the few completed clinical trials of CAR-T, a low number of Black patients with multiple myeloma were enrolled.^3,4,10^ Teclistimab, a T-cell–redirecting bispecific antibody that targets both CD3 expressed on T cells and B-cell maturation antigen expressed on the surface of multiple myeloma cells, enrolled only 12.7% Black patients.^13^ This was also evident in the trials that supported approval of idecabtagene vicleucel (6%) and ciltacabtagene autoleucel (18%).^3,4^

### Limitations

This study had several limitations. Because this was a cross-sectional study based on data available in ClinicalTrials.gov, we cannot establish specific factors that led to limited study centers in specific states. We analyzed our study based on racial and ethnic distribution in various counties. It is plausible that some patients may live in close proximity to a county of an open clinical trial but may be considered to have limited access. Our analysis did not explore if studies were planned at specific locations with no subsequent trial opening and any factors that may result in lack of equitable trial access. Nevertheless, to our knowledge, our study is the first to report on disparities in access to clinical trials of bispecific antibodies and CAR-T products that are showing promising clinical activity in patients with multiple myeloma.

## Conclusions

The geographic distribution of clinical trials for CAR-T and bispecific antibodies may be exacerbating disparities in multiple myeloma that Black patients experience. The National Cancer Institute effort to connect underrepresented populations to clinical trials is laudable, but will have limited success if most ongoing trials do not include locations in counties with high proportions of Black residents. The use of the least acceptable race-specific percentage for enrollment of Black patients in clinical trials, adjusted for the percentage of Black individuals in that state, is a potential solution that can be implemented.^12^

## Notes

Supplement. **eTable.** Characteristics Chimeric Antigen Receptor–T Cells Therapy and Bispecific Antibodies Clinical Trials in the US
