# Supplementary material for: Geographic and Racial Disparities in Access to Chimeric Antigen Receptor–T Cells and Bispecific Antibodies Trials for Multiple Myeloma
Source: JAMA Netw Open. 2022 Aug 26;5(8):e2228877. doi: 10.1001/jamanetworkopen.2022.28877 (PMC9419017; doi:10.1001/jamanetworkopen.2022.28877)
Supplement: Supplement. — eTable. Characteristics Chimeric Antigen Receptor–T Cells Therapy and Bispecific Antibodies Clinical Trials in the US [file jamanetwopen-e2228877-s001.pdf]

Supplemental Online Content

Alqazaqi R, Schinke C, Thanendrarajan S, et al. Geographic and racial disparities in access to chimeric antigen receptor–T cells and bispecific antibodies trials for multiple myeloma. *JAMA Netw Open.* 2022;5(8):e2228877. doi:10.1001/jamanetworkopen.2022.28877

eTable. Characteristics chimeric antigen receptor–T cells therapy and bispecific antibodies clinical trials in the US

This supplemental material has been provided by the authors to give readers additional information about their work.

© 2022 Alqazaqi R et al. *JAMA Network Open.*

eTable 1. Characteristics Chimeric Antigen Receptor T Cells Therapy and Bispecific Antibodies Clinical Trials in the US

State	Black or African American (%)	Black or African American (n)	Counties with open trials (n)	Black or African American in the counties with open trials n (%)	Study sites (n)	Open trials (n)	Transplant centers (n)	Number of ASCT done for MM 2015-2019
WASHINGTON DC	41.4	285810	0	N/A	0	0	1	132
MISSISSIPPI	36.6	1084481	0	N/A	0	0	1	133
LOUISIANA	31.4	1464023	1	117892 (8%)	1	2	3	349
GEORGIA	31	3320513	2	842797 (25%)	4	17	4	1703
MARYLAND	29.5	1820472	2	791348 (43.4%)	5	15	7	651
ALABAMA	25.8	1296162	1	281326 (21.7%)	1	10	3	552
SOUTH CAROLINA	25	1280531	4	166132 (12.9%)	5	1	3	468
DELAWARE	22	218899	0	N/A	0	0	1	80
NORTH CAROLINA	21	2140217	4	552240 (25.8%)	8	21	5	1526
VIRGINIA	18.6	1607581	2	118011 (7.3%)	2	3	4	632
TENNESSEE	15.8	1092948	1	173092 (15.8%)	4	20	8	939
FLORIDA	15.1	3246381	6	1755491 (54%)	11	21	8	2053
ARKANSAS	15.1	453783	1	143548 (31.6%)	1	3	1	1283
NEW YORK	14.8	2986172	7	1378815 (46.1%)	14	37	13	3008
ILLINOIS	14.1	1808271	2	1278878 (70.7%)	4	19	9	1964
MICHIGAN	13.7	1376579	2	717601 (52.1%)	5	15	5	1259
NEW JERSEY	13.1	1219770	4	212157 (17.3%)	7	19	2	1249
OHIO	12.5	1478781	2	670666 (45.3%)	4	7	6	1342
TEXAS	12.2	3552997	4	1743820 (49%)	9	30	11	3121
MISSOURI	11.4	699840	1	129814 (18.5%)	1	8	2	845
CONNECTICUT	10.8	388675	1	118933 (30.5%)	1	3	1	245
PENNSYLVANIA	10.9	1423169	2	794140 (55.8%)	4	18	9	1924

© 2022 Alqazaqi R et al. *JAMA Network Open.*

NEVADA	9.8	304739	0	N/A	0	0	N/A	0
INDIANA	9.6	648513	1	268694 (41.4%)	3	7	2	675
KENTUCKY	8	362417	2	216986 (59.8%)	3	5	2	252
OKLAHOMA	7	289961	1	120320 (41.4%)	1	3	3	192
MASSACHUSETTS	7	494029	1	145566 (29.4%)	4	24	7	1078
MINNESOTA	7	398434	1	11113 (2.7%)	4	19	2	1400
WISCONSIN	6	376256	2	276336 (73.4%)	5	22	4	1076
KANSAS	5.7	168809	1	28781 (17%)	1	6	2	662
RHODE ISLAND	5.7	62168	0	N/A	0	0	1	29
CALIFORNIA	5.7	2237044	6	1056908 (47.2%)	10	33	11	3264
NEBRASKA	4.9	96535	2	64721 (67%)	1	2	4	297
ARIZONA	4.7	339150	2	300423 (88.5%)	4	14	5	521
IOWA	4.1	131972	1	12847 (9.7%)	1	4	1	405
COLORADO	4.1	234828	2	81501 (34.7%)	3	10	2	705
WASHINGTON	4	307565	1	151468 (49.2%)	5	15	3	594
WEST VIRGINIA	3.7	65813	0	N/A	0	0	1	111
NORTH DEKOTA	3.4	26783	0	N/A	0	0	N/A	0
ALASKA	3	21898	0	N/A	0	0	N/A	0
NEW MEXICO	2.2	45904	0	N/A	0	0	1	24
SOUTH DAKOTA	2	17842	0	N/A	0	0	1	82
OREGON	2	82655	1	45370 (54.8%)	2	5	3	482
MAINE	1.9	25752	0	N/A	0	0	N/A	0
HAWAII	1.6	23417	0	N/A	0	0	1	0
NEW HAMPSHIRE	1.5	20127	0	N/A	0	0	1	98
VERMONT	1.4	9034	0	N/A	0	0	1	39
UTAH	1.2	40058	1	23474 (58.6%)	1	5	2	425
WYOMING	0.9	5232	0	N/A	0	0	N/A	0
IDAHO	0.9	15726	0	N/A	0	0	1	81
MONTANA	0.5	5484	0	N/A	0	0	1	30

ASCT; autologous stem cell transplantation, **MM**; multiple myeloma, **N/A**; not applicable

© 2022 Alqazaqi R et al. *JAMA Network Open.*
